# Lycopene as Medicine: Unlocking the Therapeutic Power of a Bioactive Carotenoid

**DOI:** 10.1021/acsomega.6c01929

**Published:** 2026-05-05

**Authors:** Farid Hajareh Haghighi, Roya Binaymotlagh, Paula Stefana Pintilei, Laura Chronopoulou, Cleofe Palocci

**Affiliations:** † Department of Chemistry, 9311 Sapienza University of Rome, Piazzale Aldo Moro 5, Rome 00185, Italy; ‡ Research Center for Applied Sciences to the Safeguard of Environment and Cultural Heritage (CIABC), Sapienza University of Rome, Piazzale Aldo Moro 5, Rome 00185, Italy

## Abstract

Lycopene, a naturally occurring carotenoid predominantly found in tomatoes and other red fruits, has attracted growing scientific interest due to its potent bioactive properties and wide-ranging therapeutic potential. This mini-review delves into the multifaceted role of lycopene in human health, with particular emphasis on its antioxidant, anti-inflammatory, and metabolic regulatory functions. We present its applications across a broad spectrum of conditions, including cardiovascular disease, cancer, metabolic disorders, reproductive health, neurodegenerative diseases, eye health, and viral infections. Special attention is given to recent advancements in nanoformulated lycopene delivery systems designed to address challenges in bioavailability and enhance targeted therapeutic efficacy. Additionally, we discuss safety profiles, dosage considerations, and regulatory frameworks relevant to lycopene-based interventions. Despite encouraging findings, significant gaps remain in long-term clinical data and the standardization of formulations. This review highlights lycopene’s promise as a versatile therapeutic agent and advocates for continued interdisciplinary research to facilitate its translation into effective clinical applications.

## Graphical abstract

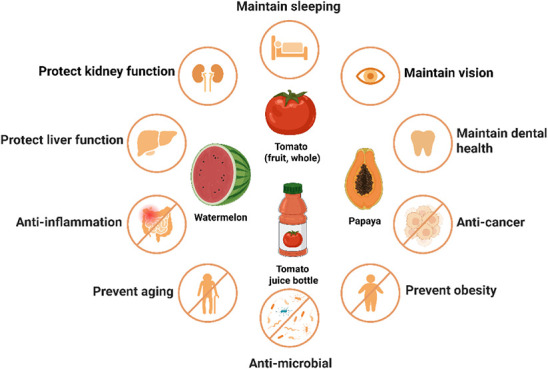

## 1 Introduction

Lycopene is a red carotenoid pigment known for its strong antioxidant properties and health benefits. Structurally, it is a linear hydrocarbon (C_40_H_56_) with 13 double bonds, 11 of which are conjugated.^1^ (Figure 1).

**Figure 1:**
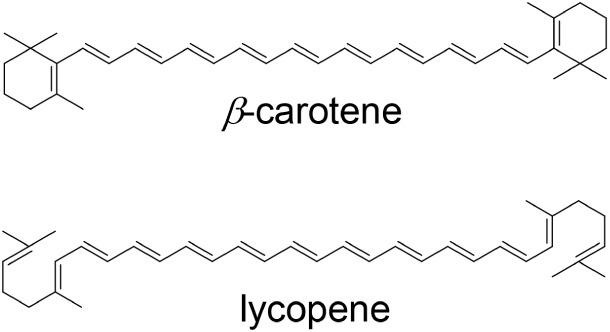
Chemical structure of lycopene and β-carotene.

It exists predominantly in the all-*trans* isomeric form in raw foods, but thermal processing (e.g., cooking tomatoes) converts it into *cis*-isomers, which are more bioavailable and readily absorbed in human tissues. Dietary sources of lycopene are abundant and include tomatoes, watermelon, pink grapefruit, papaya, guava, and rosehips. Among these, tomatoes and tomato-based products (such as sauces, juices, and pastes) account for over 80% of lycopene intake in Western diets.^2^ As one of the most potent dietary antioxidants, its molecular structure enables efficient quenching of singlet oxygen and scavenging of free radicals, thereby reducing oxidative stress that damages lipids, proteins, and DNA. In addition to antioxidant activity, lycopene exerts strong anti-inflammatory effects by modulating cytokine profiles and key signaling pathways, counteracting the chronic inflammation underlying atherosclerosis, diabetes, cancer, and autoimmune disorders.^3^ It also influences hormonal balance and metabolic regulation, particularly in obesity, metabolic syndrome, and type 2 diabetes, where insulin resistance and lipid dysregulation are central features.^4^ Beyond these roles, lycopene interacts with transcription factors and signaling cascades that govern cell proliferation, apoptosis, differentiation, and immune responses, underscoring its broad impact on cellular function. Together, these mechanisms highlight lycopene’s multifaceted bioactivity in maintaining health and reducing the risk of noncommunicable diseases.^5^

## 2 Therapeutic and Preventive Roles of Lycopene

### 2.1 Cardiometabolic Health

Lycopene exerts significant cardioprotective effects by improving lipid metabolism and supporting vascular function. It reduces low-density lipoprotein (LDL) cholesterol by binding to LDL particles and preventing oxidative modification, a key step in atherosclerosis development. Lycopene also lowers total cholesterol and triglycerides by inhibiting HMG-CoA (3-hydroxy-3-methylglutaryl-coenzyme A) reductase and upregulating hepatic LDL receptors, thereby enhancing lipid clearance. While high-density lipoprotein (HDL) levels are not markedly increased, its functionality improves through enhanced antioxidant capacity and reverse cholesterol transport. Beyond lipid regulation, lycopene combats atherosclerosis and endothelial dysfunction by reducing vascular inflammation, preserving nitric oxide bioavailability, and improving vasodilation. These combined actions reflect a systems-level modulation of cardiovascular health, encompassing lipid profiles, blood pressure, and vascular integrity. Regular consumption of lycopene-rich foods—such as tomatoes, watermelon, and guava—within diets rich in antioxidants and anti-inflammatory compounds, like the Mediterranean diet, has been consistently associated with reduced cardiovascular morbidity and mortality.^6^

### 2.2 Cancer Prevention and Adjunctive Therapy

Lycopene exhibits strong anticancer activity by suppressing uncontrolled cell proliferation, inducing apoptosis, and modulating key signaling pathways. It halts tumor growth through cell cycle arrest at G1/S and G2/M checkpoints, downregulating cyclins and CDKs while upregulating inhibitors such as p21 and p27. Apoptosis is promoted via mitochondrial and death receptor pathways, with increased levels of Bcl-2-associated X protein (Bax), reduced levels of B-cell lymphoma 2 (Bcl-2), and activation of caspases. Lycopene also inhibits nuclearfactor kappa-light-chain-enhancer of activated B cells (NF-κB), lowering pro-inflammatory cytokines, and impedes angiogenesis and metastasis by downregulating vascularendothelialgrowthfactor (VEGF) and matrixmetalloproteinases (MMPs). Beyond these intrinsic effects, lycopene enhances conventional cancer therapies. It sensitizes tumor cells to chemotherapy agents, mitigates oxidative stress in normal tissues, and supports radiation therapy by reducing ROS-induced damage (ROS: reactive oxygen species). In hormone-sensitive cancers, it improves outcomes by modulating receptor signaling, while its immunomodulatory actions—such as boosting natural killer cell activity—may complement immunotherapy. Collectively, these mechanisms highlight lycopene’s dual role as both a direct anticancer agent and a synergistic adjunct to established treatments.^7^

### 2.3 Reproductive and Hormonal Health

Lycopene plays a multifaceted role in combating metabolic disorders such as obesity, type 2 diabetes, and metabolic syndrome—conditions driven by oxidative stress, inflammation, and insulin resistance. Its antioxidant and anti-inflammatory properties reduce ROS and pro-inflammatory cytokines, improving adipose tissue function. Lycopene also modulates adipokines by normalizing leptin and enhancing adiponectin, thereby improving insulin sensitivity and reducing inflammation. It suppresses adipogenesis through downregulation of transcription factors like peroxisome proliferator-activated receptor γ (PPARγ) and CCAAT/Enhancer-Binding Protein α (C/EBPα), while favorably influencing lipid metabolism by lowering triglycerides and LDL cholesterol and enhancing HDL functionality. At the hormonal level, lycopene enhances insulin signaling via insulinreceptorsubstrate (IRS) activation andphosphoinositide3-kinase/protein kinase B (PKB) (PI3K/Akt) pathways, reduces IRS-1 phosphorylation, and activates Sirtuin 1 (SIRT1) to improve mitochondrial efficiency and metabolic flexibility. These combined effects contribute to better glucose uptake, energy balance, and protection against age-related insulin resistance. Collectively, lycopene emerges as a promising dietary compound for regulating metabolism and reducing the burden of obesity and diabetes.^8^

Lycopene supports fertility in both men and women by counteracting oxidative stress and restoring hormonal balance. In males, supplementation improves sperm concentration, motility, and morphology by protecting spermatozoa from ROS damage and enhancing Leydig cell function, which sustains testosterone production. It also safeguards the hypothalamic–pituitary–gonadal axis, helping regulatefollicle-stimulatinghormone (FSH) andluteinizinghormone (LH) for effective spermatogenesis.^1^ In females, lycopene protects ovarian follicles from oxidative injury, improves oocyte quality, and balances estrogen and progesterone levels that are essential for ovulation and endometrial receptivity. Its anti-inflammatory and antiandrogenic effects benefit conditions such as endometriosis and polycysticovarysyndrome (PCOS), where it enhances insulin sensitivity, lowers excess androgens, and promotes regular cycles. Evidence from animal and in vitro studies further supports its role in improving ovarian morphology, follicular development, and granulosa cell function.^9^ Collectively, lycopene emerges as a promising modulator of reproductive health through its antioxidant, anti-inflammatory, and endocrine-regulating activities.^10^

### 2.4 Neurological Protection

The brain’s high oxygen demand and lipid content make it vulnerable to oxidative stress, a key driver of neuronal death, inflammation, and neurodegenerative diseases such as Alzheimer’s (AD) and Parkinson’s (PD). Lycopene protects neural tissues by neutralizing free radicals, enhancing endogenous antioxidant enzymes, and reducing lipid peroxidation, DNA damage, and protein oxidation. It activates PI3K/Akt and nuclear factor erythroid 2–related factor 2 (Nrf2) pathways to promote cell survival, suppress apoptosis, and strengthen antioxidant defenses, while inhibiting nuclear Factor kappa-light-chain-enhancer of activated B cells (NF-κB) signaling and supporting brain-derivedneurotrophicfactor (BDNF) expression for neuronal repair and synaptic maintenance. In AD, lycopene reduces amyloid-β aggregation, preserves mitochondrial integrity, and downregulates pro-inflammatory cytokines, thereby limiting neuronal apoptosis and improving synaptic function. In PD, it attenuates dopamine oxidation, restores mitochondrial activity, and reduces apoptosis through the modulation of Bax/Bcl-2 and caspase-3. Preclinical studies show improved cognition, motor coordination, and reduced neuroinflammation, while observational data link higher lycopene levels to lower AD mortality. Its ability to cross the blood–brain barrier (BBB) and accumulate in neural tissues underscores lycopene’s promise as a dietary neuroprotective agent and adjunct therapy for neurodegenerative disorders.^11^

### 2.5 Immune and Infectious Disease Modulation

Lycopene demonstrates promising antiviral and immunomodulatory properties, making it a potential adjunct in managing viral infections such as SARS-CoV-2, HIV, HPV, and hepatitis C. Its antiviral effects include disrupting viral entry, suppressing RNA synthesis, and modulating host signaling pathways such as NF-κB and mitogen-activatedproteinkinase (MAPK), thereby reducing replication efficiency and protecting host cells from oxidative damage. In COVID-19, lycopene may help prevent cytokine storms by lowering Interleukin-6 (IL-6) and tumornecrosisfactor α (TNF-α), while preserving lung tissue integrity and reducing oxidative stress. Equally important are its immunomodulatory actions, which fine-tune both innate and adaptive immunity. Lycopene enhances macrophage phagocytosis, supports NK cell activity, and promotes balanced T and B cell responses while reducing excessive cytokine release through NF-κB and MAPK inhibition and strengthening antioxidant defenses via Nrf2 activation. Evidence from preclinical and clinical studies suggests that lycopene supplementation can restore immune balance, lower inflammation, and improve recovery from respiratory infections. Safe at doses of 15–45 mg/day, and with bioavailability enhanced by dietary fats and heat processing, lycopene emerges as a safe, multifunctional compound with potential to support antiviral defense and immune resilience.^12^

### 2.6 Lycopene and Eye Health: A Vision-Saving Antioxidant

Lycopene has emerged as a powerful ally in preserving eye health. Unlike other carotenoids such as β-carotene, lycopene does not convert into vitamin A, but its potent antioxidant properties make it uniquely effective in combating oxidative stress—a major contributor to age-related eye diseases. The eyes, constantly exposed to light and environmental toxins, are particularly vulnerable. Lycopene helps neutralize these free radicals, protecting delicate eye tissues such as the retina and lens from degeneration. This protection is especially critical in preventing conditions such as age-related macular degeneration (AMD) and cataracts, both of which are leading causes of vision loss in older adults. Liu et al.^13^ investigated the protective effects of lycopene against corneal endothelial damage, a condition driven by oxidative stress from environmental factors and genetic mutations. While lycopene is recognized as a potent natural antioxidant, its role in corneal endothelial health has remained unclear. Using an oxidative stress-induced injury model in a human corneal endothelial cell line (B4G12 cells) and a genetically engineered mouse model of Fuchs’ endothelial corneal dystrophy (FECD), the study demonstrated that lycopene effectively preserved corneal endothelial cells and slowed disease progression. Mechanistically, lycopene upregulated P62 and activated autophagy, promoting Keap1 degradation, facilitating Nrf2 nuclear translocation, and enhancing the expression of downstream antioxidant proteins. These findings highlight lycopene’s potential as a nonsurgical therapeutic approach for preventing or mitigating corneal endothelial damage. Alabdulmunem^14^ investigated the protective effects of lycopene in the context of diabetic retinopathy, a leading cause of vision loss among individuals with diabetes. Oxidative stress has been widely recognized as a central contributor to the pathogenesis of both diabetes and its retinal complications. Epidemiological evidence indicates that diets rich in carotenoid-containing fruits and vegetables are associated with a reduced risk of diabetes-related complications, including retinopathy. In this study, the antioxidant potential of lycopene was evaluated using D407 retinal pigment epithelial (RPE) cells through incorporation studies and the MTT (3-(4,5-dimethylthiazol-2-yl)-2,5-diphenyltetrazolium bromide) cell cytotoxicity assay. Lycopene demonstrated robust antioxidant activity *in vitro* and exhibited no cytotoxic effects on Retinal Pigment Epithelium (RPE) cells. These findings suggest that lycopene may serve as a promising agent to slow or prevent the progression of diabetic retinopathy by mitigating oxidative stress while maintaining cellular safety. Recent studies have shown that lycopene supplementation can enhance macular pigment density, which shields the retina from harmful blue light and supports visual acuity and contrast sensitivity. Additionally, lycopene’s anti-inflammatory properties help reduce chronic inflammation in the eyes, which is linked to conditions like uveitis and dry eye syndrome.^15^ By inhibiting pro-inflammatory cytokines, lycopene not only alleviates symptoms but also contributes to long-term eye resilience.^16^ Compared to other eye-supporting carotenoids such as lutein and zeaxanthin, lycopene offers complementary benefits. While lutein and zeaxanthin concentrate in the macula, lycopene’s broader antioxidant activity supports the entire ocular system. This makes it a valuable addition to any eye health regimen, through either diet or supplementation. Incorporating lycopene-rich foods into daily meals is a simple yet effective strategy.^17^ Table 1 summarizes the mechanisms of action, advantages, and existing limitations of lycopene for various diseases.

**Table 1:** Mechanisms, Advantages, and Limitations of Lycopene across Disease Areas

Disease Area	Primary Mechanisms of Action	Key Advantages	Existing Limitations	Refs
Cardiovascular disease	• Antioxidant reduction of LDL oxidation	• Nonpharmacological cardioprotective option	• Variable bioavailability	^6^
	• Modulation of lipid metabolism (↓ LDL, ↑ HDL)	• Supports vascular health	• Dose–response inconsistency in human trials	
	• Improvement of endothelial function	• Synergistic with statins and lifestyle interventions	• Limited long-term clinical data	
	• Anti-inflammatory effects on vascular tissue			
Cancer prevention & adjuncttherapy	• ROS scavenging	• Broad anticancer potential	• Mechanisms differ across cancer types	^7^
	• Modulation of cell cycle and apoptosis pathways	• Low toxicity	• Inconsistent clinical outcomes	
	• Inhibition of IGF-1 signaling	• Can enhance effects of chemotherapy	• Limited tumor-targeted delivery	
	• Antiangiogenic effects			
Metabolic disorders (diabetes, obesity)	• Improvement of insulin sensitivity	• Supports glucose homeostasis	• Human evidence still limited	^8^
	• Reduction of oxidative stress in pancreatic β-cells	• Potential adjunct to metabolic therapies	• Effects vary by formulation and dose	
	• Anti-inflammatory modulation of adipokines			
Reproductive health	• Protection of sperm DNA from oxidative damage	• Improves sperm quality and motility	• Small sample sizes in studies	^1^
	• Hormonal modulation	• Potential fertility benefits	• Lack of standardized dosing	
	• Anti-inflammatory effects in reproductive tissues			
Neurological disorders	• Antioxidant protection of neurons	• Potential neuroprotective agent	• Poor blood-brain barrier penetration	^11^
	• Reduction of neuroinflammation	• May support cognitive health	• Limited clinical trials in humans	
	• Modulation of mitochondrial function			
Immune & antiviral effects	• Modulation of cytokine production	• Broad immunomodulatory potential	• Mostly preclinical evidence	^13^
	• Enhancement of innate immune responses	• Supports antiviral defense	• Mechanisms not fully defined	
	• Reduction of viral-induced oxidative stress			
Eye health	• Protection against oxidative damage in retina	• May reduce risk of macular degeneration	• Limited clinical trials	^13^,^14^
	• Reduction of inflammation	• Synergistic with other carotenoids	• Bioavailability highly formulation-dependent	
	• Support of photoreceptor integrity			

## 3 Advances in Lycopene Delivery and Bioavailability

### 3.1 Challenges in Bioavailability and Stability

Lycopene faces several bioavailability challenges that limit its therapeutic potential. One major issue is its poor water solubility; lycopene is highly lipophilic and virtually insoluble in water, which hampers its absorption in the gastrointestinal tract. In aqueous environments, it tends to aggregate and crystallize, further reducing its bioaccessibility.^18^ Additionally, lycopene is extremely sensitive to environmental stressors, such as light (leading to photooxidation), oxygen (causing autoxidation), and heat (triggering thermal isomerization). These factors collectively contribute to the degradation of lycopene, resulting in the loss of color, potency, and biological activity during processing and storage.^19^ Another complication arises from isomerization: while the all-*trans* form is predominant in raw foods, it is less bioavailable compared to the *cis*-isomers. However, although food processing can increase the proportion of *cis*-isomers and thereby improve absorption, these forms tend to be less chemically stable. Biological barriers also impede lycopene’s effectiveness, including enzymatic degradation in the gastrointestinal tract—particularly by β-carotene oxygenases—and limited transport across the intestinal epithelium due to its poor solubility and the lack of specific carrier proteins. Together, these factors significantly constrain lycopene’s systemic availability and therapeutic efficacy.^20^

Nanoencapsulation has emerged as a powerful strategy to overcome lycopene’s physicochemical limitations. These systems protect lycopene from degradation, enhance solubility, and improve bioavailability.^21^ Nanoformulations have emerged as an effective strategy to improve the stability, bioavailability, and delivery of lycopene. Various encapsulation materials are employed, including natural polymers such as chitosan, alginate, and gelatin; synthetic polymers like PLGA (poly­(lactic-*co*-glycolic acid)), PEG (polyethylene glycol), and PCL (polycaprolactone); and proteins such as whey protein isolate, sodium caseinate, and pea protein.^22^ Surfactants such as Tween 20 and lecithin are also commonly used to stabilize formulations. One of the major advantages of nanoencapsulation is its role in enhancing lycopene stability. The encapsulation matrix acts as a physical barrier, protecting lycopene from environmental stressors such as oxygen, light, and heat.^23^ Additionally, emulsifiers and surfactants help stabilize the nanodispersion by preventing particle aggregation, and coencapsulation with antioxidants like vitamin E can further enhance protection. Several optimization parameters are critical in nanoformulation design: smaller particle sizes tend to improve intestinal absorption; zeta potential values greater than ±30 mV indicate strong electrostatic repulsion and good colloidal stability; encapsulation efficiency reflects the percentage of lycopene successfully enclosed within the carrier; and the release profile determines how lycopene is gradually delivered over time, which is crucial for maintaining therapeutic levels.

Targeted delivery systems for lycopene are designed to enhance therapeutic efficacy by directing the compound to specific tissues or cells, thereby maximizing its biological effects while minimizing systemic side effects. These systems utilize a range of targeting strategies. Passive targeting takes advantage of the enhanced permeability and retention (EPR) effect commonly seen in tumor tissues, allowing nanoparticles to accumulate more readily in these areas. In contrast, active targeting employs specific ligands—such as folic acid or antibodies—that bind to receptors overexpressed on target cells.^24^ Additionally, stimuli-responsive systems have been developed, enabling lycopene release in response to environmental triggers such as pH changes, temperature shifts, or the presence of specific enzymes. To further improve therapeutic control, controlled-release mechanisms are incorporated, including diffusion-controlled systems (where lycopene gradually diffuses through a matrix), erosion-controlled systems (where matrix degradation leads to release), and swelling-controlled systems (in which environmental changes cause the matrix to expand and release its contents).^25^ One example is lycopene-loaded PLGA nanoparticles, which have demonstrated 3- to 9-fold higher bioaccessibility than free lycopene, sustained release over a 12-day period, and non-Fickian diffusion kinetics—suggesting a complex and efficient release profile.^26^ Moreover, organ-specific delivery of lycopene using nanocarriers has been successfully achieved, targeting the liver for hepatoprotective effects, the prostate for anticancer applications, the brain for neuroprotection, and the skin for anti-inflammatory and photoprotective benefits.^27^ Table 2 compares key parameters such as encapsulation efficiency, release profiles, and targeting capabilities across these different systems.

**Table 2:** Comparison of Nanodelivery Systems for Lycopene

Delivery System	Encapsulation Efficiency (EE%)	Release Profile	Targeting Capability	Stability	Key Limitations	Refs
Liposomes	Moderate–high (50–90%)	Sustained release; rate depends on lipid composition	Passive targeting; can be surface-modified for active targeting	Sensitive to oxidation and leakage; stability improved with cholesterol	Prone to degradation; short shelf life; batch variability	^28^
PLGA nanoparticles	High (70–95%)	Controlled, slow release due to polymer degradation	Can be functionalized with ligands (e.g., folate, peptides)	Excellent stability; protects lycopene from light/heat	Requires organic solvents; potential burst release; cost of scale-up	^29^
Solid lipid nanoparticles (SLNs)	Moderate (40–80%)	Sustained release; matrix limits diffusion	Limited intrinsic targeting; modifiable with surface ligands	High physical stability; good protection against oxidation	Lower loading capacity; risk of polymorphic transitions	^30^
Nanostructured lipid carriers (NLCs)	High (70–95%)	Improved sustained release vs SLNs	Modifiable for active targeting	Very stable; high loading due to mixed lipid matrix	Complex formulation; potential long-term storage issues	^31^
Nanoemulsions	Moderate (40–70%)	Rapid to moderate release depending on surfactant	No inherent targeting; can incorporate targeting ligands	Good kinetic stability; enhances solubility	Surfactant-related toxicity; instability under extreme pH	^32^
Polymeric micelles	High (60–90%)	Sustained release; responsive to pH or temperature	Good for tumor targeting (EPR effect); ligand modification possible	High stability in aqueous environments	Limited loading for highly crystalline lycopene; dilution instability	^33^
Cyclodextrin complexes	Low–moderate (20–50%)	Fast release; weak host–guest interactions	No targeting unless conjugated	Improves solubility; moderate stability	Low encapsulation; weak protection from degradation	^34^
Chitosan nanoparticles	Moderate–high (50–85%)	Sustained release; pH-responsive	Mucoadhesive; good for oral and nasal targeting	Good stability; protects against oxidation	Limited loading; variability in chitosan quality	^35^
Metal–organic frameworks (MOFs)	Very high (>90%)	Highly controlled release; tunable porosity	Potential for ligand-based targeting	Excellent structural stability	Safety concerns; limited food-grade MOFs; regulatory uncertainty	^36^

### 3.2 Clinical Relevance of Enhanced Formulations

Enhanced lycopene formulations have shown promising results in both preclinical and clinical studies. Human trials have demonstrated that lycopene nanoemulsions can significantly improve plasma concentrations and the levels of antioxidant markers. Additionally, encapsulated lycopene has been observed to reduce prostate-specific antigen (PSA) levels in men with benign prostatic hyperplasia, while liposomal lycopene formulations exhibit superior skin penetration and help mitigate UV-induced damage.^37,38^ From a pharmacokinetic perspective, nanoformulations enhance the absorption rate, increase the peak plasma concentration (*C*_max_), and improve the area under the curve (AUC), with controlled release systems maintaining therapeutic levels over extended periods. At the molecular level, lycopene interacts with various targets, including nuclear receptors such as PPARγ and RAR, G protein-coupled receptors (GPCRs), and ion channels. Computational pharmacology studies have further identified mitogen-activated protein kinase kinase 2 (MAP2K2), sodium voltage-gated channel alpha subunit 2 (SCN2A), and tripartite motif containing 24 (TRIM24) as high-affinity targets for lycopene. In terms of safety, most lycopene nanoformulations are biocompatible and nontoxic, with long-term studies reporting no adverse effects at therapeutic doses. However, regulatory approval for these formulations depends on their specific type and intended application.^39^

Smart delivery systems for lycopene, such as thermoresponsive polymers such as Poly­(*N*-isopropylacrylamide)–poly­(ethylene glycol) (PNIPAAM–PEG), enable temperature-triggered release, while pH-sensitive carriers target the tumor microenvironment more effectively. Hybrid systems that combine lycopene with other bioactive compounds, including curcumin and resveratrol, offer synergistic therapeutic effects through coencapsulation strategies aimed at multitarget therapy.^40^ Personalized nutrition approaches are emerging, tailoring lycopene formulations based on individual genetic factors that influence absorption and integrating these formulations into functional foods and beverages. From a regulatory and commercial perspective, the market for lycopene-based supplements and fortified foods is expanding, but there remains a critical need for standardized protocols in encapsulation methods and stability testing.^41^ Additionally, clinical trials are essential to validate the efficacy and safety of these products across diverse populations. In conclusion, while lycopene’s therapeutic potential is vast, its clinical utility depends on overcoming challenges related to bioavailability and stability. Advances in nanoformulations, encapsulation technologies, and targeted delivery systems have markedly improved lycopene’s pharmacokinetic profile and therapeutic efficacy, facilitating its application in disease prevention and treatment, paving the way for its role in personalized medicine and functional nutrition.

## 4 Safety, Dosage, and Regulatory Considerations

### 4.1 Toxicological Profile and Safety Margins

Lycopene is generally considered safe when consumed through food sources and exhibits a favorable toxicological profile. It is nonmutagenic, noncarcinogenic, and does not accumulate in tissues to toxic levels. Both animal and human toxicity studies consistently show a low risk, even at high doses.^42^ Regarding acute toxicity, no lethal dose (LD_50_) has been established due to lycopene’s low toxicity; animal studies administering doses up to 1000 mg/kg body weight reported no adverse effects. Chronic exposure studies in rats and mice also revealed no significant organ damage or carcinogenicity with prolonged supplementation. While some safety data sheets mention a possible risk of impaired fertility or harm to the unborn child at extremely high concentrations, such effects have not been observed at dietary or therapeutic levels.^43^ Lycopene may act as a moderate irritant to the skin and eyes in its pure form, but no allergic reactions have been reported from dietary lycopene, although rare hypersensitivity to tomato-based products may occur.^44^ The observed safe level (OSL) for adults is up to 75 mg per day, with wide safety margins as therapeutic doses remain well below toxic thresholds. Additionally, lycopene is nonhazardous for transport and does not require special handling under normal conditions.^45^

### 4.2 Dietary Intake and Therapeutic Dosing

Lycopene dietary intake varies widely depending on individual diet, with average consumption in Western diets ranging from 3 to 10 mg per day.^46^ Rich sources of lycopene include raw tomatoes, which contain approximately 3.1 mg per 100 g, tomato sauce with 33–68 mg per 100 g, and ketchup at about 3.3 mg per tablespoon.^47^ Other fruits, such as watermelon, pink grapefruit, and guava, provide between 3 and 8 mg per serving. Therapeutic doses of lycopene, often delivered via supplements, are typically higher. General supplementation of 15–45 mg per day for up to six months is considered safe. In the context of prostate cancer support, doses between 10 and 30 mg per day—or up to 43 mg per day from tomato-based products—have shown benefits in stabilizing PSA levels.^48^ For cardiovascular and antioxidant support, 10–20 mg of daily doses has been found to improve lipid profiles and oxidative stress markers, while doses of 10–15 mg per day enhance skin photoprotection by increasing UV resistance and reducing erythema. Because lycopene is lipophilic, its absorption improves when consumed with dietary fats, and processed tomato products such as paste and juice offer better bioavailability than raw tomatoes due to isomerization into more absorbable *cis*-forms. Supplement forms include softgels, capsules, and nanoemulsions, which may further enhance absorption. No serious adverse effects have been reported at therapeutic doses, though mild gastrointestinal symptoms such as nausea, diarrhea, or bloating may occur in sensitive individuals. Excessive intake can lead to lycopenodermia, a harmless condition characterized by orange discoloration of the skin.

### 4.3 Regulatory Approvals and Guidelines

Lycopene is widely recognized and regulated as a safe food additive across multiple regions. In the United States, the FDA (U.S. Food and Drug Administration) approves lycopene as a color additive under 21 CFR §73.585 for use in foods, with tomato lycopene extract and concentrate exempt from certification due to their established safety profile. It can be used broadly in foods, except where standards of identity prohibit added color, and must comply with Good Manufacturing Practices (GMP) and labeling requirements outlined in §70.25.^49^ In the European Union, lycopene is listed as E160d and permitted as a food colorant in products such as beverages, dairy, confectionery, and supplements, with maximum levels varying by product category.^50^ The European Food Safety Authority (EFSA) has reviewed lycopene’s safety and concluded that it poses no safety concerns at typical intake levels.^51^ Internationally, Codex Alimentarius and the World Health Organization recognize lycopene as a safe food additive and antioxidant, including it in global food standards for coloring and fortification, and have not established an acceptable daily intake (ADI) due to its low toxicity. In countries such as Japan and China, lycopene is approved for use in functional foods and supplements, regulated under local food safety laws and labeling requirements, and commonly marketed for skin health, antioxidant support, and cardiovascular benefits. Regarding labeling and health claims, any assertions must be supported by clinical evidence, with lycopene supplements often promoted for heart health, prostate support, skin protection, and antioxidant defense. Labels are required to specify the source, such as tomato extract, dosage, and any contraindications.^52^ In conclusion, lycopene is a well-tolerated, widely consumed carotenoid with a robust safety profile and broad regulatory acceptance. While dietary intake typically provides modest levels, therapeutic supplementation offers enhanced benefits for cardiovascular, reproductive, and skin health. Advances in formulation have improved its bioavailability, and global regulatory agencies have approved its use in foods and supplements with minimal restrictions. As research continues to explore lycopene’s therapeutic potential, its safety, versatility, and regulatory clarity make it a valuable component of modern nutrition and preventive medicine.

## 5 Current Limitations and Research Gaps

### 5.1 Inconsistencies in Clinical Outcomes

Despite promising preclinical data and epidemiological associations, translating these benefits into consistent clinical outcomes has proven to be challenging. Several limitations and research gaps hinder lycopene’s full integration into evidence-based therapeutic protocols. These include variability in clinical results, lack of standardized formulations and dosing, and a scarcity of long-term human trials.^53^ While numerous studies suggest that lycopene may reduce the risk of chronic diseases such as prostate cancer, cardiovascular disease, and metabolic syndrome, clinical outcomes remain inconsistent. Some trials report significant improvements in biomarkers, whereas others show negligible or no effects. These discrepancies arise from multiple sources. Heterogeneous study designs—differing in population demographics, sample sizes, and measured end points—make cross-study comparisons difficult, and the source of lycopene varies widely across studies, with interventions ranging from raw tomatoes and tomato paste to supplements and synthetic formulations, each with distinct bioavailability profiles. The duration of intervention is another critical variable; short-term studies may not fully capture lycopene’s therapeutic potential.^54^ Dietary confounders, including the baseline diet and lifestyle habits, are often insufficiently controlled and may obscure true effects. A notable example is prostate health research, where some clinical trials have shown reductions in PSA levels and tumor volume following lycopene supplementation, while others report no significant changes despite increased serum lycopene concentrations.^55^ These inconsistencies may reflect differences in formulation, dosage, and patient risk profiles, as well as biological variability in absorption, metabolism, and tissue distribution. Genetic polymorphisms affecting carotenoid transport and metabolism may further modulate individual responses to supplementation.^56^ Additionally, variability in biomarker selection—ranging from oxidative stress markers to inflammatory cytokines and lipid parameters—limits comparability across studies and may not always align with lycopene’s primary mechanisms of action. To address these issues, future clinical studies should adopt standardized, high-bioavailability formulations; harmonize dosing regimens; employ unified and mechanistically relevant biomarker panels; and stratify participants based on baseline lycopene status, metabolic health, and genetic background. Incorporating pharmacokinetic assessments, extending study durations, and improving control of dietary and lifestyle confounders will further strengthen the reliability of clinical findings and help clarify lycopene’s true therapeutic potential.

### 5.2 Need for Standardized Formulations

There is a notable lack of uniformity in how lycopene is administered across studies with various formulations including capsules, softgels, emulsions, and food-based matrices. These differ significantly in isomer composition (*trans vs*. *cis* forms), carrier oils and excipients, particle size, and delivery systems, making it difficult to compare outcomes across clinical trials.^57^ Dosing further complicates the landscape: typical dietary intake ranges from 3 to 15 mg/day in Western diets, while supplemental doses can vary widely from 10 to 75 mg per day. Currently, there is no established Recommended Dietary Allowance (RDA) for lycopene.^58^ Moreover, bioavailability may plateau at doses between 10 and 30 mg, beyond which absorption shows diminishing returns. Analytical standardization presents another challenge. Although methods such as reverse-phase high-performance liquid chromatography (RP-HPLC) and spectrophotometry are used to quantify lycopene in supplements and foods, inconsistencies in extraction and measurement techniques hinder reliable dose–response evaluations.^59^ The formulation of lycopene significantly influences its bioavailability. Processed tomato products like paste and sauce, for example, offer superior absorption compared with raw sources due to heat-induced isomerization. Emerging technologies such as nanoformulations and encapsulation methods, including liposomes and PLGA nanoparticles, have demonstrated improved stability and absorption. However, these advanced delivery systems remain unstandardized across clinical studies, further complicating the interpretation of efficacy data.^60^

### 5.3 Limited Long-Term Human Trials

Most human studies of lycopene focus on short-term interventions lasting weeks to a few months, leaving long-term safety, efficacy, and disease-modifying effects largely unexplored. Many trials suffer from small sample sizes, limiting their power to detect meaningful clinical outcomes, and few include follow-up periods to assess sustained benefits or potential adverse effects over years. Additionally, most studies rely on surrogate markers—such as oxidative stress levels or PSA—rather than hard clinical outcomes like disease incidence or mortality.^61^ In cancer prevention trials, particularly for prostate cancer, systematic reviews reveal mixed results, underscoring the need for long-term studies to clarify lycopene’s potential to reduce cancer incidence or progression.^62^ Preclinical research suggests neuroprotective effects of lycopene, but human trials remain scarce and short-term, with longitudinal studies needed to determine its role in cognitive aging and neurodegenerative diseases such as AD and PD. In cardiovascular health, lycopene has been shown in short-term studies to lower LDL cholesterol and improve endothelial function; however, long-term trials are necessary to establish its impact on major cardiovascular events such as heart attacks and strokes. Overall, while lycopene holds significant promise as a bioactive compound with broad health benefits, clinical translation is hindered by inconsistent trial outcomes driven by variability in study design, dosing, and formulation; a lack of standardized delivery systems and analytical methods; and insufficient long-term human data to support disease prevention claims. To advance lycopene from a promising phytonutrient to a validated therapeutic agent, rigorous large-scale randomized controlled trials using standardized formulations, consensus on optimal dosing and bioavailability-enhancing strategies, and longitudinal studies assessing sustained efficacy and safety are essential.

## 6 Future Directions

### 6.1 Emerging Technologies in Lycopene Delivery

Its full therapeutic potential remains underutilized due to challenges in bioavailability, formulation, and clinical translation. As research advances, new technologies and strategies are reshaping how lycopene is delivered, personalized, and integrated into preventive healthcare. As mentioned above, lycopene is highly lipophilic and unstable when exposed to light, heat, and oxygen, which limits its effectiveness. To address these challenges, researchers are developing advanced delivery systems that protect lycopene and enhance its absorption.^63^ Key innovations include nanoemulsions—oil-in-water emulsions with droplet sizes under 200 nm that improve solubility and gastrointestinal uptake—and liposomes, which are phospholipid vesicles that encapsulate lycopene, offering biocompatibility and controlled release. Solid lipid nanoparticles (SLNs) provide high loading capacity and protect lycopene from oxidation, while biodegradable polymeric nanoparticles such as PLGA enable targeted delivery and sustained release.^64^ These technologies not only increase bioavailability but also allow site-specific delivery to organs such as the liver, prostate, or brain, enabling disease-specific applications. In parallel, green extraction methods are advancing lycopene production with higher purity and sustainability. Techniques such as pulsed electric fields (PEF) enhance extraction yield and antioxidant activity with minimal energy use; ultrasound-assisted extraction improves recovery while reducing solvent consumption; and supercritical CO_2_ extraction offers a solvent-free, efficient process suitable for pharmaceutical-grade lycopene.^65^ These approaches align with green chemistry principles and support clean-label product development. Moreover, smart controlled-release systems are being designed to release lycopene in response to physiological triggers, such as pH-sensitive carriers that activate in acidic tumor microenvironments, thermoresponsive polymers that release lycopene at elevated temperatures found in inflamed tissues, and enzyme-responsive systems that activate in the presence of disease-specific enzymes.^66^ These smart delivery platforms enable precision dosing, minimize side effects, and enhance therapeutic outcomes.

### 6.2 Personalized Medicine Approaches

Personalized medicine increasingly leverages individual genetic, metabolic, and lifestyle data to tailor interventions, and lycopene’s role in this paradigm is gaining attention, particularly in chronic disease prevention.^54^ Genetic factors such as polymorphisms in carotenoid metabolism genes like β-Carotene 15,15’-Monooxygenase 1 (BCMO1) and Scavenger Receptor Class B Member 1 (SCARB1) influence lycopene absorption and tissue distribution, meaning individuals with reduced bioavailability may require higher doses or enhanced formulations. Metabolic conditions including obesity, diabetes, and aging are associated with lower plasma lycopene levels and impaired tissue uptake, highlighting the need for personalized dosing strategies to compensate for these metabolic impairments.^67^ Advances in computational pharmacology have identified lycopene’s molecular targets—such as MAP2K2, SCN2A, and TRIM24—which show variable expression across tissues. Network pharmacology approaches further reveal that lycopene modulates interconnected signaling pathways involving nuclear receptors, G protein-coupled receptors (GPCRs), and ion channels, supporting targeted supplementation based on individual disease risk and molecular profiles. Additionally, emerging evidence suggests that lycopene can beneficially influence the gut microbiota by enhancing populations of probiotics such as *Lactobacillus* and *Bifidobacterium* while reducing pro-inflammatory species linked to metabolic and immune disorders. This microbiome modulation opens the door for personalized lycopene interventions aimed at optimizing gut–brain axis function, immune support, and metabolic health.^68^

### 6.3 Integration into Preventive Healthcare Strategies

Lycopene is increasingly incorporated into functional foods, such as enriched yogurts, cereals, and beverages, providing convenient delivery methods that promote disease prevention and overall wellness. It is also utilized in sports nutrition products for its antioxidant and anti-inflammatory benefits, supporting lifestyle-based prevention, particularly for cardiovascular and metabolic disorders.^69^ Beyond individual use, lycopene’s preventive potential is being explored in population health contexts, where it has demonstrated improvements in endothelial function and reductions in LDL oxidation relevant to cardiovascular disease, associations with lower risks of prostate, breast, and digestive cancers, and protective effects on skin against UV damage as well as support for bone density. Integrating lycopene into dietary guidelines and public health campaigns offers promising avenues to reduce the disease burden and healthcare costs. Clinically, although lycopene is not yet mainstream, its use is advancing as an adjunct in prostate cancer management and cardiovascular risk reduction, included in personalized supplement regimens for high-risk individuals, and monitored via serum levels to assess antioxidant status and dietary adequacy.^70^ Future protocols may incorporate lycopene in preventive panels, especially for aging populations and those experiencing chronic inflammation. Overall, lycopene is undergoing a transition from a dietary antioxidant to a precision health tool. Emerging delivery technologies like nanoencapsulation and smart carriers are improving its stability, bioavailability, and therapeutic reach, while personalized medicine approaches tailor its use based on genetics, metabolism, and microbiome profiles. As research continues to refine these innovations, lycopene is poised to become a cornerstone of next-generation nutrition and preventive medicine, effectively bridging the gap between food and pharmaceuticals.

## 7 Conclusion

Lycopene, a naturally occurring carotenoid abundant in tomatoes, watermelon, pink grapefruit, and other red fruits, has emerged as a powerful bioactive compound with diverse therapeutic applications. Its unique molecular structure, containing 11 conjugated double bonds, confers exceptional antioxidant capacity, enabling it to neutralize reactive oxygen species more effectively than other carotenoids, such as β-carotene and α-tocopherol. This antioxidant potency underpins lycopene’s broad biological effects across multiple organ systems and disease states. Preclinical and clinical studies demonstrate its efficacy in cancer prevention and therapy by inhibiting cell proliferation, inducing apoptosis, and modulating signaling pathways such as phosphoinositide 3-kinase/protein kinase B/mechanistic target of rapamycin (PI3K/Akt/mTOR), NF-κB, and insulin-like growth factor-1 (IGF-1), particularly in prostate, breast, colon, and skin cancers. Lycopene also provides cardiovascular protection by lowering LDL cholesterol, improving endothelial function, and reducing atherosclerotic plaque formation, while its metabolic benefits include enhancing insulin sensitivity, modulating adipokines, and reducing oxidative stress in metabolic syndrome, obesity, and type 2 diabetes. Neuroprotective effects are evident in its ability to shield neurons from oxidative damage, preserve mitochondrial function, and mitigate neurodegenerative diseases such as Alzheimer’s and Parkinson’s. In reproductive health, lycopene improves sperm quality, regulates sex hormones, and protects reproductive tissues from oxidative and inflammatory damage. Emerging evidence further highlights its antiviral activity, including the inhibition of viral replication such as SARS-CoV-2 and modulation of immune responses. These multifaceted effects position lycopene as a compelling candidate for integrative disease prevention and adjunctive therapy, with its safety profile, natural origin, and dietary availability enhancing its appeal as a functional nutrient.

Despite this promise, clinical translation faces challenges due to lycopene’s poor water solubility, chemical instability, and variable bioavailability. Advances in formulation science—including nanoencapsulation, liposomal delivery, and polymeric carriers—have improved its pharmacokinetic properties, while computational pharmacology and systems biology approaches have identified high-affinity molecular targets such as MAP2K2, SCN2A, and TRIM24, supporting its role as a multitarget therapeutic agent. Translational potential is further strengthened by tissue-specific delivery systems, controlled-release technologies, and synergistic combinations with other bioactives or conventional therapies, bridging the gap between bench and bedside. Looking ahead, future research should prioritize precision nutrition approaches that integrate genomics, metabolomics, and microbiome profiling to identify the individuals most likely to benefit from lycopene-based interventions. Such personalized strategies may help explain interindividual variability in absorption and response, enabling tailored dosing and formulation selection. Additionally, multifunctional combination therapies—pairing lycopene with other antioxidants, phytochemicals, or standard pharmacotherapies—represent a promising direction for enhancing efficacy through complementary mechanisms of action.

To fully realize lycopene’s therapeutic potential, standardized formulations, optimized dosing regimens, and robust long-term clinical trials remain essential. Interdisciplinary collaboration will be critical: pharmacologists and formulation scientists must develop stable, bioavailable delivery systems; molecular biologists and computational scientists should continue elucidating mechanisms and molecular targets; clinicians and epidemiologists need to conduct well-controlled, longitudinal studies; and nutritionists and public health experts should integrate lycopene into dietary guidelines and preventive health strategies. Research priorities include the development of validated biomarkers for absorption and therapeutic response, exploration of gene–nutrient interactions, and evaluation of lycopene within precision health frameworks that combine lifestyle, dietary, and molecular data. Ultimately, lycopene represents a paradigm shift in the use of dietary compounds for therapeutic purposes. Its potent antioxidant and anti-inflammatory properties, coupled with disease-specific efficacy, make it a valuable tool against chronic illness. While challenges remain, the convergence of technological innovation, molecular insight, and personalized healthcare strategies offers a clear path forward. With rigorous research and translational development, lycopene can evolve from a promising phytonutrient into a cornerstone of integrative and precision medicine, bridging nutrition, pharmacology, and public health for a healthier future.
